# Supplementary material for: Theta beta ratio in attention deficit hyperactivity disorder using a multiverse analysis
Source: eLife. 2026 Jul 9;15:RP111114. doi: 10.7554/eLife.111114 (PMC13349384; doi:10.7554/eLife.111114)
Supplement: Peer review history

# Editor report: eLife Assessment

**DOI:** 10.7554/eLife.111114.3.sa0

**Authors:** Markus Ploner

**Affiliations:** Department of Neurology and TUM-Neuroimaging Center, TUM School of Medicine and Health, Technical University of Munich (TUM) Germany

**Keywords:** Exceptional, Important

This manuscript addresses an **important** question in clinical neuroscience: the use of the theta/beta ratio as a biomarker of attention deficit hyperactivity disorder (ADHD). The study takes an **exceptional** "multiverse" analysis approach to show that aperiodic activity differences between healthy controls and people with ADHD are driving the apparent theta/beta ratio differences. From a neuroscientific perspective, this is a critical finding because it has a major impact on guiding research on the diagnosis and treatment of ADHD.

# Referee report: Reviewer #1 (Public review)

**DOI:** 10.7554/eLife.111114.3.sa1

[Editors' note: this version has been assessed by the Reviewing Editor without further input from the original reviewers. The authors have addressed the comments raised in the previous round of review.]

Summary:

The authors address whether theta/beta ratio /TBR can be used as a clinical biomarker for ADHD.

Strengths:

The data were acquired independently from 2 separate datasets, and there are sufficient subjects for adequate statistical power. The authors applied up-to-date EEG data preprocessing, state-of-the-art feature extraction, and statistical analyses, using a multiverse approach. By testing and comparing all meaningful approaches, defined a priori in the previous meta-analysis, the author convincingly demonstrates that TBR cannot be used as a clinical biomarker, and previous positive results can be explained by interactions between different factors (alpha peak frequency, aperiodic component, age).

Weaknesses:

There are no apparent issues with data, separate datasets, large sample sizes, and state-of-the-art data analysis.

# Referee report: Reviewer #2 (Public review)

**DOI:** 10.7554/eLife.111114.3.sa2

Summary:

This manuscript examines whether the theta-beta ratio as derived from EEG data relates to ADHD diagnoses. To do so, it performs a multiverse analysis across a large number of analytical choices, applied to a large EEG dataset, and corroborated in an additional validation set. The results overall show that the TBR is not a reliable indicator of ADHD diagnosis. In discussing the patterns of results across analytical choices, the authors also demonstrate some key points about what appears to be driving the ratio measures, noting that significant results appear to be driven by choices regarding aperiodic-correction and the use of individualized alpha frequencies, suggesting TBR measures can be affected by these features rather than reflecting theta and/or beta activity.

Strengths:

This manuscript addresses a clearly posed and important question in the literature, addressing a longstanding discussion on the relationship between TBR and ADHD, and uses a large dataset and an expansive analysis approach to provide a definitive answer. The strengths of the approach allow for a clear answer, providing a notable contribution to the field.

Weaknesses:

I find no notable weaknesses in the current manuscript nor any major issues that I think challenge the key findings of this manuscript.

# Referee report: Reviewer #3 (Public review)

**DOI:** 10.7554/eLife.111114.3.sa3

Summary:

In this manuscript, Strzelczyk, Vetsch, and Langer tackle an incredibly important question in clinical neuroscience: the use of the theta/beta ratio as a biomarker of attention deficit hyperactivity disorder (ADHD). The theta/beta ratio is argued to be so reliable as an ADHD biomarker that, in the United States, the Food and Drug Administration has approved its use as a biomarker for ADHD diagnosis. However, there is mounting evidence that the theta/beta ratio is likely not really measuring the relative power between two oscillations - the theta rhythm and the beta rhythm - but rather reflects differences in a singular, non-oscillatory aperiodic process. In this very convincing study, Strzelczyk and colleagues take a "multiverse" analysis approach to show that aperiodic activity differences between healthy controls and people with ADHD are driving the apparent theta/beta ratio differences. While in a vacuum, where a measure is a measure and if it's related to a diagnosis it's still useful no matter what, this distinction might not seem important, from a neuroscientific perspective this is a critical distinction, because the ratio between two oscillations has fundamentally very different underlying physiological mechanisms than aperiodic differences, and this framing has a major impact on guiding research on the diagnosis and treatment of ADHD.

Strengths:

While smaller studies and analyses have already hinted at similar results as shown here, the current study's multiverse analysis approach is comprehensive, convincing, and very well done. The large sample size of 1,499 participants is very impressive, as is the use of an independent validation sample of 381 participants.

Overall, the technical and statistical aspects are very well done: the multiverse approach, the validation set, the resampling methods, and even the shiny apps. The authors should be applauded for being so thorough and making their data and analyses publicly accessible.

Weaknesses:

To be clear, I see no breaking weaknesses in the theoretical foundations, methods, statistical analyses, or interpretations.

# Author comment: Author response

**DOI:** 10.7554/eLife.111114.3.sa4

**Authors:** Dawid Strzelczyk, Andrea Vetsch, Nicolas Langer

**Affiliations:** University of Zurich Zürich Switzerland; University of Zurich Zürich Switzerland; University of Zurich Zurich Switzerland

The following is the authors’ response to the original reviews.

**Reviewer #1 (Public review):**

Summary:

The authors address whether theta/beta ratio /TBR can be used as a clinical biomarker for ADHD.

Strengths:

The data were acquired independently from 2 separate datasets, and there are sufficient subjects for adequate statistical power. The authors applied up-to-date EEG data preprocessing, state-of-the-art feature extraction, and statistical analyses, using a multiverse approach. By testing and comparing all meaningful approaches, defined a priori in the previous meta-analysis, the author convincingly demonstrates that TBR cannot be used as a clinical biomarker, and previous positive results can be explained by interactions between different factors (alpha peak frequency, aperiodic component, age).

Weaknesses:

There are no apparent issues with data, separate datasets, large sample sizes, and state-of-the-art data analysis.

We thank Reviewer #1 for their positive evaluation of our manuscript and for the constructive recommendations. The reviewer did not raise additional comments requiring a point-by-point response beyond the recommendations addressed below.

**Reviewer #2 (Public review):**

Summary:

This manuscript examines whether the theta-beta ratio as derived from EEG data relates to ADHD diagnoses. To do so, it performs a multiverse analysis across a large number of analytical choices, applied to a large EEG dataset, and corroborated in an additional validation set. The results overall show that the TBR is not a reliable indicator of ADHD diagnosis. In discussing the patterns of results across analytical choices, the authors also demonstrate some key points about what appears to be driving the ratio measures, noting that significant results appear to be driven by choices regarding aperiodic-correction and the use of individualized alpha frequencies, suggesting TBR measures can be affected by these features rather than reflecting theta and/or beta activity.

Strengths:

This manuscript addresses a clearly posed and important question in the literature, addressing a longstanding discussion on the relationship between TBR and ADHD, and uses a large dataset and an expansive analysis approach to provide a definitive answer. The strengths of the approach allow for a clear answer, providing a notable contribution to the field.

Weaknesses:

I find no notable weaknesses in the current manuscript nor any major issues that I think challenge the key findings of this manuscript.

We thank Reviewer #2 for their positive evaluation of our manuscript and for the constructive recommendations. The reviewer did not raise additional comments requiring a point-by-point response beyond the recommendations addressed below.

**Reviewer #3 (Public review):**

Summary:

In this manuscript, Strzelczyk, Vetsch, and Langer tackle an incredibly important question in clinical neuroscience: the use of the theta/beta ratio as a biomarker of attention deficit hyperactivity disorder (ADHD). The theta/beta ratio is argued to be so reliable as an ADHD biomarker that, in the United States, the Food and Drug Administration has approved its use as a biomarker for ADHD diagnosis. However, there is mounting evidence that the theta/beta ratio is likely not really measuring the relative power between two oscillations - the theta rhythm and the beta rhythm - but rather reflects differences in a singular, non-oscillatory aperiodic process. In this very convincing study, Strzelczyk and colleagues take a "multiverse" analysis approach to show that aperiodic activity differences between healthy controls and people with ADHD are driving the apparent theta/beta ratio differences. While in a vacuum, where a measure is a measure and if it's related to a diagnosis it's still useful no matter what, this distinction might not seem important, from a neuroscientific perspective this is a critical distinction, because the ratio between two oscillations has fundamentally very different underlying physiological mechanisms than aperiodic differences, and this framing has a major impact on guiding research on the diagnosis and treatment of ADHD.

Strengths:

While smaller studies and analyses have already hinted at similar results as shown here, the current study's multiverse analysis approach is comprehensive, convincing, and very well done. The large sample size of 1,499 participants is very impressive, as is the use of an independent validation sample of 381 participants.

Overall, the technical and statistical aspects are very well done: the multiverse approach, the validation set, the resampling methods, and even the shiny apps. The authors should be applauded for being so thorough and making their data and analyses publicly accessible.

Weaknesses:

To be clear, I see no breaking weaknesses in the theoretical foundations, methods, statistical analyses, or interpretations. All of my recommendations below are for the sake of clarity, which I believe is especially important because this is such an important paper that many people should read.

Comments:

(1) Some figures are mislabeled. For example, Supplementary Figure 1 says (C) are scalp topographies, but those are (A), while (C) shows power spectra, but it's unclear what (C) is. I assume it's only the aperiodic part of the spectrum (oscillations removed)? But it would be better to plot on a log-log scale if so. In fact, I recommend showing all spectra on a log-log scale.

The reviewer is correct that the figure legend was mislabeled. Panel (A) shows the scalp topographies, panel (B) shows the 1/f-uncorrected power spectra, and panel (C) shows the reconstructed aperiodic signal with oscillations removed. We have corrected the figure legend accordingly. In addition, the power spectra and the reconstructed aperiodic signal are now plotted on log-log scales to improve readability and interpretability.

(2) Supplementary Figure 6 is also mislabeled, saying (A) shows age (it does not) and so on.

We thank the reviewer for noticing this error. We have revised the figure legend so that the panel descriptions now match the displayed plots.

(3) In Supplementary Figure 7, is (B) the aperiodic-removed spectrum? The authors are very inconsistent with what they're showing in these spectral plots, and not actually explaining what they're showing: raw spectra, semi-logged or not, aperiodic-removed or oscillations-removed, etc.

Panel (B) in Supplementary Figure 7 shows the aperiodic-adjusted spectrum. We have now corrected the figure labeling and revised the figure legend to explicitly state what is shown in each panel.

(4) For the HBN data, it is said that, "electrode impedances were kept below 40 kΩ, lower than EGI's standard recommendation of 50 (Net Station Acquisition Technical Manual)." For the validation data: "... electrode impedances were maintained below 5 kΩ." These are big impedance threshold differences. Of course, these recommendations differ by recording system, the use of active electrodes, and so on. But such differences can certainly influence signal-to-noise. The fact that the results are so consistent between them is a strength that perhaps should be explicitly called out.

We appreciate the reviewer’s suggestion. We now explicitly state in the discussion section that the consistency of the results across datasets with different EEG systems and impedance thresholds strengthens the generalizability of the findings. The revised text reads as follows:

“Our multiverse results thus converge with this broader literature, providing further evidence that TBR lacks the reliability and discriminative validity required for clinical utility. Beyond methodological convergence across analytical frameworks, the consistency of results across two datasets differing substantially in EEG recording systems and impedance thresholds further strengthens the generalizability of these null findings, suggesting they are unlikely to reflect idiosyncrasies of a specific acquisition protocol.”

(5) The authors cite a lot of foundational / related work here, such as Finley et al, but they should also cite several other highly relevant ones:

Saad et al., "Is the Theta/Beta EEG Marker for ADHD Inherently Flawed?", J Atten Disord, 2015

Donoghue, Dominguez, Voytek, "Electrophysiological frequency band ratio measures conflate periodic and aperiodic neural activity", eNeuro, 2020

Karalunas et al., "Electroencephalogram aperiodic power spectral slope can be reliably measured and predicts ADHD risk in early development", Develop Psychobiol, 2022

Donoghue, "A systematic review of aperiodic neural activity in clinical investigations", Eur J

Neurosci 2025

We thank the reviewer for pointing us to these additional relevant references. We have added the suggested references to the revised manuscript.

**Recommendations for the authors:**

**Reviewer #1 (Recommendations for the authors):**

(1) "Multiverse analysis was conducted in RStudio (R version 4.4.1) using the multiverse package (version 0.6.1; Sarma et al., 2021). T" Ok, cool, but it would be useful to explain what it does compared to running the standard stat analysis N times.

We thank the reviewer for this helpful recommendation. We have now expanded the Methods section to clarify this point. The revised text reads as follows:

“Multiverse analysis was conducted in RStudio (R version 4.4.1) using the multiverse package (version 0.6.1; Sarma et al., 2021). The multiverse framework differs from simply repeating the same statistical analysis multiple times, because it first requires the researcher to define a structured analysis space consisting of multiple defensible analytic decisions. These decisions are then expanded into all valid combinations, with each combination representing one complete analysis specification, or “universe”, providing a transparent and reproducible record of which analytic decisions were considered and how they were combined. In addition, the package reduces the need to manually write, modify, and track separate analysis scripts for each specification, which helps avoid inconsistencies or coding errors across universes. The results can then be extracted and summarized across the full set of universes to evaluate whether the conclusions are robust across reasonable analytic alternatives or depend on specific combinations of choices.”

(2) I may have missed it, but how many subjects per group do you end up with after all the cleaning (not what is in Table 1, but like in each dataset you describe how many got removed at each step, so we are left wondering the final numbers).

We thank the reviewer for pointing this out. The final group sizes after all cleaning and exclusion steps were not described in the original manuscript. We have therefore revised Table 1 so that it now reports only the remaining participants included in the final analyses after all exclusions were applied. The revised table shows the final sample sizes separately for the HC (N = 228), ADHD-Combined (N = 429), and ADHD-Inattentive (n = 465) groups, together with the corresponding demographic and clinical characteristics. We have also revised the accompanying text in the result section 3. 1. 1. The same changes were applied to the validation sample, which is reported in the Supplement.

(3) Missing reference in my opinion. In the discussion, the sentence "as both oscillatory and aperiodic contributions vary systematically across the lifespan" could do with a reference or two about that

We have now added references showing that developmental changes in EEG spectra involve both periodic/oscillatory and aperiodic components. The revised text reads as follows:

“These dynamics may account for the recurring Age ’ IAF interactions observed in our multiverse analyses, as both oscillatory and aperiodic contributions vary systematically across the lifespan (Merkin et al. 2023; Tröndle et al. 2022; Tröndle et al. 2021; McSweeney et al. 2023; Hill et al. 2022; Stanyard et al. 2024).”

(4) Now the big one: this is a cool visualization, and beta estimates from linear modeling do tell us the strength, BUT I would like to see raw effect sizes. It could be in a table or text, to go with the discussion. What was the theta, alpha, beta power raw or adjusted in each group, what about the aperiodic component - even maybe some violin plots to show canonical vs individual - my point is I am convinced from the frequency analysis since an entire subspace become significant and your interpretation that this is spurious is satisfactory but showing that this subspace as tiny effect sizes driven by interactions would be even more convincing in my opinion.

To complement the regression coefficients from the multiverse models, we now additionally report descriptive standardized effect sizes across representative analytical subspaces. Specifically, we grouped analytical paths according to frequency band definition (IAF-relative vs canonical) and spectral representation (aperiodic signal, 1/f-uncorrected power, and aperiodic-adjusted power). Within each subspace, we computed Cohen’s d values for theta power, beta power, and TBR between ADHD and healthy control groups across all corresponding analytical paths.

To visualize the distribution of effects across analytical paths, we added violin plots with overlaid individual paths and mean effect sizes with 95% confidence intervals. Importantly, even in subspaces where interaction effects frequently emerged in the multiverse analysis, the corresponding descriptive group differences remained small, supporting our interpretation that the observed significant effects are driven by subtle interactions and analytical choices rather than large underlying group differences.

The added text in the Results 3. 1. 4. reads as follows:

“To complement the regression coefficients from the multiverse models, we additionally examined descriptive standardized effect sizes across representative analytical subspaces. Analytical paths were grouped according to frequency band definition (IAF-relative vs. canonical) and spectral representation (aperiodic signal, 1/f-uncorrected power, and aperiodic-adjusted power). Within each subspace, Cohen's d was computed for theta power, beta power, and TBR for both the HC vs. ADHD-Inattentive and HC vs. ADHD-Combined comparisons. To visualize the distribution of effect sizes across the analytical space, violin plots were constructed with each data point representing the Cohen's d value of a single analytical specification (Figure 8). Across all subspaces and outcome measures, Cohen's d values were small for both comparisons, including subspaces in which interaction effects frequently reached statistical significance in the multiverse analysis. This pattern indicates that even where the multiverse revealed reliable significant effects, the underlying group differences in theta power, beta power, and TBR remained small in magnitude. These findings support the interpretation that the significant interactions observed across analytical specifications are driven by subtle moderation effects and analytical choices rather than large, robust group differences in neural activity.”

**Reviewer #2 (Recommendations for the authors):**

(1) As a minor clarification, the manuscript could specify if the calculation of aperiodic-adjusted power values was done as subtraction with linear or log power values.

The aperiodic-adjusted power values were computed by subtracting the aperiodic fit from the observed power spectrum in log10 power space. Specifically, both the observed power spectrum and the estimated aperiodic component were log10-transformed, and the aperiodic-adjusted signal was obtained as the difference between these two quantities. The result was then transformed back to linear scale. We have clarified this in the revised manuscript. The revised text reads as follows:

“The aperiodic component was reconstructed based on its fitted parameters and subtracted from the total power spectrum in log10 power space, resulting in an aperiodic-adjusted, 1/f-corrected power spectrum. The resulting values were then transformed back to linear scale and therefore represent power relative to the estimated aperiodic background.”

(2) The last section of the abstract is a bit repetitive in stating the main finding of what drives the TBR, and this could be edited/condensed.

We agree that the final part of the abstract repeated the main interpretation regarding the role of aperiodic activity and IAF. We have therefore condensed this section to avoid redundancy while preserving the central conclusion. The revised text reads as follows:

Across the multiverse, we found that group differences in TBR were highly contingent on analytical choices, with no evidence for robust main effects of diagnosis, indicating no reliable differences between healthy controls, ADHD-inattentive, and ADHD-combined subtypes. Instead, significant effects emerged primarily as interactions with age and individual alpha frequency (IAF), particularly when TBR was derived from aperiodic-uncorrected power or from the aperiodic signal itself. These interaction patterns replicated across both independent samples and were observed using both categorical and dimensional definitions of ADHD. Together, these findings indicate that previously reported TBR effects are largely driven by variability in aperiodic activity and IAF rather than genuine differences in oscillatory theta-beta dynamics. Our results challenge the interpretation of TBR as a reliable standalone biomarker for ADHD and underscore the importance of multiverse approaches for evaluating candidate neurobiological markers in heterogeneous clinical populations.

(3) As a minor literature note, the finding that ratio measures often largely reflect aperiodic activity rather than oscillatory theta and/or beta per se activity is consistent with a previous (non-clinical) investigation of band ratio measures in a previous report that should perhaps be cited as relevant prior work:

Donoghue, T., Dominguez, J., & Voytek, B. (2020). Electrophysiological Frequency Band Ratio Measures Conflate Periodic and Aperiodic Neural Activity. eNeuro, 7(6),ENEURO.0192-20.2020. https://doi.org/10.1523/ENEURO.0192-20.2020

We appreciate the reviewer’s suggestion. We have added this reference to the Discussion section, where we interpret the observed TBR effects as reflecting variability in the aperiodic background rather than genuine differences in oscillatory theta-beta dynamics. The revised text reads as follows:

“These results suggest that apparent TBR differences may reflect properties of the aperiodic background signal interacting with individual variability in IAF rather than true oscillatory theta or beta activity. This interpretation is consistent with previous work showing that electrophysiological frequency-band ratio measures can conflate periodic and aperiodic neural activity, such that apparent changes in theta/beta or other band ratios may partly reflect changes in the aperiodic spectral component rather than narrowband oscillatory activity (Donoghue et al., 2020).”

(4) In Figure 3, it may be useful to highlight the theta and beta ranges in panel B.

We considered highlighting the theta and beta ranges in Figure 3B, but decided against it. In the multiverse analysis, theta and beta were defined using both canonical frequency bands and IAF-relative bands. The IAF-relative bands differ across participants, therefore marking only the canonical ranges could give the impression that these were the only frequency definitions used in the analyses. We therefore kept the spectra unmarked.

(5) In Figure 5 (and other figures following this motif), it may be useful to color the significant results as green or red based on direction, to match Figure 4.

We have updated Figure 5 and the corresponding figures so that significant positive effects are shown in green and significant negative effects are shown in red, matching the color scheme used in Figure 4.

**Reviewer #3 (Recommendations for the authors):**

(1) P10, L30: "Individualized bands were centered on the IAF, defined as theta = IAF-6 Hz to IAF-4 Hz"; why is theta defined using such a narrow, 2 Hz band here, when canonical theta is usually defined as a 4 Hz wide, 4-8 Hz band?

The individualized theta band was chosen to follow the IAF-based frequency-band framework proposed by the seminal work of Wolfgang Klimesch (1999, 2012), rather than to reproduce the width of the canonical 4-8 Hz theta band. In this framework, frequency bands are defined relative to each participant’s individual alpha frequency. Theta is defined as the range from IAF-6 Hz to IAF-4 Hz, while lower alpha occupies the range closer to the individual alpha peak. The narrower individualized theta band is therefore intended to reduce overlap with lower-alpha activity and to account for inter-individual and developmental differences in alpha peak frequency. The 2020 guidelines from the International Federation of Clinical Neurophysiology (IFCN) reaffirmed Klimesch’s division of the alpha and theta bands (Babiloni, 2020). We have explained the frequency bands selection in more detail in the manuscript. The revised text reads as follows in Methods 2. 5. 7. Extraction of power for statistical analyses:

The selection of these frequency bands is grounded in the seminal work of Wolfgang Klimesch (1999), who demonstrated that the alpha band can be divided into distinct lower and upper sub-bands. The lower alpha band extends up to 4 Hz below the IAF, covering a broader range of approximately 3.5 to 4 Hz, while the upper alpha band, which lies above the IAF, is narrower, spanning about 1 to 1.5 Hz. Klimesch also characterized the theta band as a frequency range that is approximately 2 Hz below the lower alpha band (Klimesch, 1999; Klimesch, 2012). The 2020 guidelines from the International Federation of Clinical Neurophysiology (IFCN) reaffirmed Klimesch’s division of the alpha and theta bands (Babiloni, 2020).

(2) Figure 3 and Supplementary Figure 1, 7, 8: "Electrodes highlighted on the topographies..." means just the text labels, right? It might be better to show all electrodes as black dots and highlight the others with white dots or something.

We have revised the figures to display all electrodes as black dots. In addition, we have clarified in the figure legends that the highlighted electrode labels correspond to the regions of interest used in the analyses.
